# A randomized controlled trial to evaluate an educational strategy involving community health volunteers in improving self-care in patients with chronic heart failure: Rationale, design and methodology

**DOI:** 10.1186/2193-1801-3-689

**Published:** 2014-11-25

**Authors:** Soraya Siabani, Tim Driscoll, Patricia M Davidson, Stephen R Leeder

**Affiliations:** Menzies Centre for Health Policy, School of Public Health, Medical School, The University of Sydney, Sydney, NSW Australia; School of Public Health, Kermanshah University of Medical Sciences, Kermanshah, Iran; Epidemiology and Occupational Medicine, School of Public Health, Sydney Medical School, The University of Sydney, Sydney, NSW Australia; Department of Acute and Chronic Care, The Johns Hopkins University, Baltimore, MD USA

**Keywords:** Community health workers, Chronic illnesses, Home-based education, Health education, Congestive heart failure, Self-management, Medical adherence, Adherence to treatment

## Abstract

**Background:**

Chronic heart failure (CHF) is an increasingly important health problem worldwide. Effective self-care can improve the outcomes and quality of life in patients with CHF. Acknowledging the important role of educational interventions for improving self-care, we sought to assess a new educational strategy involving community health volunteers (CHVs) that could reduce the cost and, hypothetically, increase the effectiveness of self-care education in patients with CHF.

**Methods/Design:**

In this ongoing three-arm controlled trial, approved by two human research ethics committees in Australia and Iran, 231 patients with CHF registered at a referral cardiovascular hospital in Iran were randomly allocated into three groups -trained by community health volunteers at patients’ homes, rained by formal health professionals at hospital; and a control group with no formal educational exposure. Data obtained through interviewing participants and using the Persian self-care of CHF index (pSCHFI) before and two months after interventions will be analysed using SAS and SPSS.

**Discussion:**

The results of this study may help health service systems, especially in countries with limited resources, make use of community volunteers to teach patients with CHF to develop self-care behaviors and skills, reducing the cost of care and improving CHF outcomes. Also, this home-based educational strategy using face-to-face training, if successful, may provide psychosocial supports for patients suffering from chronic illnesses.

**Trial registration number:**

ACTRN12614000788673 (Australian New Zealand Clinical Trials Registry)

## Introduction

### Background

Chronic heart failure (CHF) is an increasingly important health problem with a high mortality rate and morbidity worldwide (Go et al. 2013). Patients suffering from CHF often have a low quality of life (Pinto et al. 2011), and the majority of patients die within five years of diagnosis (Norton et al. 2011). For example, approximately 1.1 million hospitalizations for CHF cost $29 billion in 2004 in the United States (Go et al. 2013), increasing to $40 billion in 2010 and similarly in European countries (Braunschweig et al. 2011). Almost 70% of the expenditure is for hospital services (Heidenreich et al. 2011). Effective self-care behaviors can increase quality of life in patients with CHF and reduce hospitalization (Moser et al. 2012).

Self-care in CHF refers to a naturalistic decision-making process including the choice of behaviours and activities that patients use to maintain life, health and well-being. It includes a healthy lifestyle (e.g. daily exercise), adherence to a treatment regimen, symptom monitoring and seeking assistance when symptoms occur or change (Riegel et al. 2009). Evidence has underscored the importance of knowledge and self-care skills in order to accomplish effective self-care in patients with CHF (van der Wal et al. 2006; Siabani et al. 2013). Health literacy accelerates self-care confidence (Dennison et al. 2011), while lack of knowledge and self-care skills is serious obstacles for patients with CHF and their adherence to treatment (Siabani et al. 2013).

During the past decade many studies have tried and assessed innovative technologies and advanced educational methods to enhance knowledge and self-care skills in patients with CHF (Baker et al. 2011; Barnason et al. 2012; Boyde et al. 2013; Boyde et al. 2011). However, these methods often prove to be ineffective. In addition, almost all interventions have been tried in developed countries rather than in populations living in countries with limited resources. Further, the technology used in these strategies (telehealth etc.) is not available in most less-developed countries. Professional-led programs, as most of these are, require resources that developing countries cannot afford (De Geest et al. 2004). Therefore, educational programs using simple language, minimal technology and taught by dedicated non-professionals is theoretically a more feasible option. Community health workers’ help is a common phenomenon, especially in low- or middle- income countries mainly in the primary care setting (Maes 2010; Hadi 2003; Sirilak et al. 2013; Giugliani et al. 2011).

In Iran, in the past three decades, a new integrated health system has been introduced. It aims to address the Iranian population health needs, especially in rural area and underprivileged urban areas (Asaei 2014; Malekafzali 2006). Obtaining benefit from the help of community health volunteers (CHVs) locally called "*Rabetin Behdasht*" is a part of this system. CHVs are mostly females with basic education – high school or beyond – interested in helping the community health services without pay. The most important role of CHVs in Iran has been building a bridge between people who need follow-up (e.g. vaccination) and health delivery systems. Each CHV covers around 50–100 families living in her neighbourhood. The CHVs are trained after joining the health delivery system and asked to attend once a month at their local health centres to be given the cases for follow up. Evidence showed important progress in improving health indicators such as infant mortality rate across the country following their deployment (Asaei 2014).

To date, CHVs have limited their function to family planning programs and primary prevention (Giugliani et al. 2011; Malekafzali 2006). Recognizing the possibility of role of educational interventions improving self-care in Iranian patients, and the limitation of resources available for this purpose we set out to assess an innovative educational strategy involving CHVs that might reduce cost and, hypothetically, increase effectiveness in improving self-care in patients with CHF.

### Aim and objectives

The aim of this study is to assess the effectiveness of a new educational strategy using CHVs (strategy I) in improving self-care in patients with CHF, in comparison with a formal educational strategy (strategy II) and a control group receiving standard care. The study hypothesis is that CHVs are equally or more effective as professional health educators in educating patients with chronic heart failure to improve self-care skills.

#### Primary objectives

To determine baseline self-care (self-care maintenance, self-care management and self-care confidence) in patients with CHF discharged from Imam Ali hospital (Iran) from August 2010 to June 2012.

To measure the scores relating to self-care (self-care maintenance, self-care management and self-care confidence) in each of the three arms of the study, two months after applying interventions.

To compare self-care maintenance, self-care management and self-care confidence among groups I and, II, I and III, II and III.

#### Secondary objective

To explore the personal characteristics such as age and gender of study subjects.

To explore the health-related factors such as duration of CHF, number of admissions for CHF, and comorbidities (e.g. diabetes) in study subjects.

To determine the association between each personal factor (e.g. education and age) that might influence the efficacy of interventional strategies.

## Methods

### Trial design

This study is an ongoing three-arm randomised controlled trial aiming to compare a new educational strategy involving community health volunteers (CHVs) with health-professional-based educational strategy, and a control group received standard care without educational intervention. Self-care skill scales will be assessed and compared in three groups of patients with heart failure.

### Participants and setting

Two hundred thirty one patients above 18 years of age, who had been admitted to hospital at least once with CHF during last two years, but not in last two months, were recruited to participate in the study. Those who had CHF of New York Heart Association Class (NYHA) functional class II or III, and did not have cognitive problem were eligible for inclusion in this study. NYHA class IV was excluded because we excluded people with any severe mental or physical disability preventing people of self-care activities. Also, we excluded patients in class I, due to being asymptomatic, though we have not been collecting the functional status as a variable in our data collection. They were from a population of whom more than 99% are Muslim. Languages spoken in this population are Kurdish and Persian. Almost all were Caucasian and have similar cultural behaviors. Participants who met the inclusion criteria were identified in advance, using the hospitalization records, by an experienced nurse with access to the records. Subjects needed valid contact details to be included. Prior permission was obtained from the director of Imam Ali Hospital, Kermanshah, Iran. Ethics approval for the study was granted by human research ethics committees in Iran (Kermanshah University of Medical Sciences Research Ethics Committee) and Australia (University of Sydney Human Research Ethics Committee); A written informed consent for participation in the study was obtained from all participants.

### Intervention

#### Educational approaches

The intervention included two different educational approaches to training self-care information to patients diagnosed with CHF. One group was trained face-to-face at their homes by CHVs and the second group was trained by a skilled nurse and a general practitioner in three small groups at a conference room in the hospital (Imam Ali Hospital).

#### Preparing educators

Prior to the intervention ten female CHVs, aged from 27 to 53 years were trained in three workshops by a general practitioner. These subjects were selected from among fifty CHVs connected to a health delivery center (Sameno-al-aemaeh) in Kermanshah. The inclusion criteria were; an acceptable level of conversation skills, ability to speak in Persian and Kurdish, having been actively involved during the preceding year in assisting at the health center and having graduated from high school or beyond. Three one-day workshops were held to train and prepare the selected CHVs, the number and duration of workshops being based on a needs-assessment conducted at the introductory session and using the feedback from participants (CHVs) after that session.

The aim of workshops was to teach the CHVs about the content of the educational programs for patients and how to present the information. The materials were obtained from heart failure text books (Bonow et al. 2011) and related sites (Krum et al. 2011) that have been translated into simple Persian language understandable for patients, even those who might be illiterate. The preparation and simplification was conducted under supervision of a cardiologist. The information was provided in a PowerPoint presentation and in a notebook used in workshops. In addition, during workshops, tutors considered the likely questions or challenging issues that might be faced during their home visits. At the end of the workshops, using role play, each of CHVs was assessed to evaluate her ability as an educator. One of CHVs who did not have an acceptable result was removed from the list. Further, CHVs were given a contact number of a trained general practitioner to ask questions during home visits.

For the second group a nurse and general practitioner both experienced and working in cardiovascular disease and cardiovascular rehabilitation were employed. They were asked to study the notebook prepared as above. They were asked to educate participants, using their own knowledge and considering the aims and objectives of education. They were blind to the study purpose.

#### Payments

The interventions were started in parallel, though educating by CHVs lasted about two weeks because each CHV had to educate approximately seven to eight patients one-by-one and at their homes. The patients in second group –educated at hospital– were offered reimbursement for travel costs but none asked. Fees were paid to educators of this group, namely, the nurse and general practitioner. CHVs making home visits were reimbursed for travel costs. Taxi fares were paid for one of the investigators who monitored the home-based education. Two months later participants in the three groups were interviewed again at home or hospital, according to their preferences.

### Outcomes

The self-care components including self-care maintenance, self-care management and self-care confidence would be the main outcome variables of the study. These outcomes will be assessed before intervention and two months after intervention. The Persian version of the self-care chronic heart failure index V 6.2 (pSCHFI) scales is used to evaluate self-care components in three groups of study. The SCHFI uses a quantitative, ordinal, self-report and performance-rating scale (Riegel et al. 2009). Both SCHFI and pSCHFI include three scales with 22 questions–10 questions for self-maintenance, six for self-management and six for self-confidence. Each sub-scale is standardized to a possible score of zero to 100. In Persian version of SCHFI, in comparison with the original SCHFI, some grammatical changes had been made and a few words had been deleted or added, however none of those modifications did not change the meaning of the questions. For example, we inserted the phrase "How routinely do you…" at the beginning of many questions of the Self-maintenance sub-scale, instead of using only one sentence at the beginning of subscale, as used in the original SCHFI. In addition, item 4, "Do some physical activity?" was changed to "Do you routinely do your normal life activities such shopping and cleaning?" (Riegel et al. 2009; Siabani et al. 2014).

In order to obtain demographic data and personal health information- as the secondary outcomes-, an investigator-constructed questionnaire has been also administered that included 13 questions about age, sex, living status (with whom they live), smoking, co-morbidities (e.g. hypertension), duration of heart failure, and the number of times a patient had been admitted to hospital due to CHF symptoms from diagnosis CHF. This information will be used to evaluate the likely association between self-care scales and personal characteristics. Data collected by three experienced trained nurses and through interviewing have been conducted at the patient’s home or hospital, according to participant preference, before intervention and two months after intervention.

### Sample size

According to the following formula and information received from another study of a similar design (Riegel et al. 2009), it was expected the mean increase in self-care would be about 22 units (d = 22).


Hence to have a power of 90% (σ =40, d = 22, Z α/2 = 1.96, Z β =0.1) minimum 60 participants were needed for each group.

**Figure 1:**
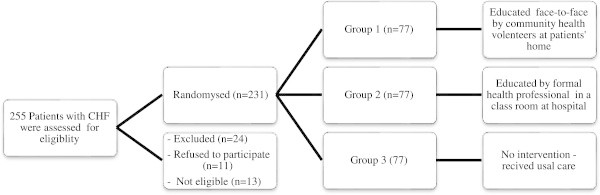
**The CONSORT flow diagram of the randomized controlled trial.**

Considering a non-response rate of 15%, the sample size for each group would be 60, and total of 180 participants. However, according expert opinions, all 231 patients who had completed the primary interviews conducted to obtain the baseline data, and agreed with remaining in the study were considered to participate in the RCT, therefore, each group would be included 77 patients (Figure 1).

### Randomization

#### Sequence generation

The random number list was generated using Microsoft Excel number generator.

#### Allocation concealment mechanism

A registered nurse working at the medical record department at Imam Ali hospital who had collected the list of eligible subjects sent them to the study leader. The study leader provided a biostatistician, who was paid by, but not involved in the study, with the list of names and subject numbers for all 231 subjects. The biostatistician randomized the list, as described above, and then sent the list of names and subject codes with the allocated group to the study leader.

#### Implementation

Subjects who met the selection criteria were contacted by a nurse working at the hospital and invited to participate in the study. Those who were available and agreed to participate were recruited to be interviewed. The interviews were conducted by two trained nurses. The completed questionnaires including patients’ names and numbers were sent to the study leader. The list of numbers of those completed a baseline interview were sent to the biostatistician to be randomized as described above. The allocation list provided to the study leader was then used to assign subjects to their group, 77 subjects allocated to group I (exposed to CHVs), 77 subjects to group II (formal health educators) and 77 patients to group III (control group).

### Blinding

Subjects were aware of where the training took place and who undertook the training and so could have been aware of the group to which they were allocated.

The first data collection occurred prior to randomization. The nurses involved in the data collection undertaken two months after the intervention were recruited to collect the data and has not have further involvement in the study. They were blinded to intervention allocation, but it is possible that the intervention allocation may have become known as a result of discussions during the data collection process.

### Statistical methods

SPSS version 21 and SAS will be used to analyse data. Using descriptive analysis for descriptive data, analytic biostatistics analysis (e.g. ANOVA, independent t-test and paired t-test) for assessing differences (between two groups, among three groups and before and after for each group) will be applied. Regression models will be used to explore associations between predicting factors (e.g. age, gender and education) and self-care scales, and also association between the effectiveness of each strategy and personal characteristics will be used. We will present frequencies and examine the expected associations between different variables to obtain answers for our research questions and hypothesis. T-tests will be used for continuous and normally distributed variables (e.g. self-care maintenance in terms of gender), chi-squared for nominal variables and other proportions (e.g. age group, sex), and multivariate generalized linear regression will be used for detecting associations between outcomes (e.g. self-care management) and explanatory variables (e.g. gender, education and disease duration). Non-parametric tests (e.g., Wilcoxon rank test and Median test) will be used when variables are found not to be normally distributed.

A summary of the interventional strategies is presented in the Table 1.

**Table 1:** **The characteristics of randomized control trial**

Group	GROUP I	GROUP II	Group III
**Intervention (education)**	Strategy I	Strategy II	Control group (Standard care)
**Educators**	CHVs	Formal health educators	-
**Strategy**	Face-to-face	Small group	-
**Duration**	2 hours	4 hours	-
**Participants**	77	77	77
**Setting**	Patients’ homes	Hospital	-
**Materials**	Simplified instruction for self-care provided by a GP and under supervision a cardiovascular specialist	Simplified instruction for self-care provided by a GP and under supervision of a cardiovascular specialist	-
**Monitoring**	Checking 25% of cases by one of investigators for intervention fidelity	An investigator attending in class without announcing	-

## Results

The results of this study will be reported once data analysis is completed.

## Conclusion

This study was designed to examine the hypothesis that CHVs through a face-to-face education program offered at patients’ homes are able to train patients with CHF to care for themselves. If true, this would be a low-cost, simple and possibly more effective educational approach in order to improve self-care in patients with CHF. This strategy, especially in countries with limited resources, can minimize inequity in health services.
